# Coupling CALPHAD Method and Entropy-Driven Design for the Development of an Advanced Lightweight High-Temperature Al-Ti-Ta Alloy

**DOI:** 10.3390/ma17215373

**Published:** 2024-11-03

**Authors:** Gourav Mundhra, Jien-Wei Yeh, B. S. Murty

**Affiliations:** 1 Department of Metallurgical and Materials Engineering, Indian Institute of Technology Madras, Chennai 600036, India; gourav.nitdurgapur.mse17@gmail.com; 2 Department of Materials Science and Engineering, National Tsing Hua University, Hsinchu 300044, Taiwan, China; 3 High Entropy Materials Centre, National Tsing Hua University, Hsinchu 300044, Taiwan, China; 4 Department of Materials Science and Metallurgical Engineering, Indian Institute of Technology Hyderabad, Telangana 502284, India

**Keywords:** advanced Al alloy, CALPHAD, alloy design, scanning electron microscopy, nanoindentation, mechanical properties

## Abstract

In this study, a new lightweight Al-Ti-Ta alloy was developed through a synergistic approach, combining CALPHAD methodology and entropy-driven design. Following compositional optimization, the Al_87.5_Ti_6.25_Ta_6.25_ (at.%) alloy was fabricated and isothermally heat-treated at 475 °C for 24 h to attain equilibrium. X-ray diffraction (XRD), scanning electron microscopy (SEM), and differential scanning calorimetry (DSC) analyses revealed a dual-phase microstructure comprising a 50 vol.% FCC matrix enriched in Al and 50 vol.% Al_3_(Ti,Ta)-type intermetallic phase (IP). Notably, the FCC phase exhibited a high-melting transition temperature of 660 °C, surpassing conventional Al-Si cast alloys. Phase-specific nanomechanical properties were evaluated using Nanoindentation. Microindentation tests demonstrated exceptional microhardness of approximately 3300 MPa. These results indicate the alloy’s superior hardness compared to conventional alloys such as Al-Si (A390), 7075 Al alloy, and CP-Ti, even exceeding Ti-64 alloy at a 15% lower density. The alloy’s stability under prolonged heat treatment at 475 °C, reflected by stable phases, microstructure, and mechanical properties, highlights its enhanced thermal stability, which can be attributed to entropy-driven phase stabilization. This study underscores the effectiveness of integrating entropy-driven design strategy with CALPHAD predictions for the accelerated development of advanced Al-based alloys.

## 1. Introduction

Amidst the dynamic evolution of the aeroengine sector, there exists a clear emphasis on pioneering lightweight materials. This imperative arises from the pressing need to bolster component reliability and adhere to progressively increasing governmental directives aimed at attaining net-zero emissions. Within this complex context, the quest for innovative lightweight alloys tailored for engines assumes paramount importance. These alloys are meticulously designed not merely to attenuate noise, mitigate greenhouse gas emissions, and curtail fuel consumption but also to enable operations at high temperatures (HT), heralding a profound leap towards sustainable aviation [1].

The front section of an aeroengine, traditionally constructed from titanium (Ti)-based alloys, accounts for approximately 70–80% of the total thrust [2]. It is well established that a lighter front section enhances propulsive efficiency [1]. Titanium alloys, particularly Ti-6Al-4V, have been favored due to their high specific strength and excellent fatigue resistance [3]. However, these alloys are costly, with a density of 4.45 g/cm^3^ [3]. Magnesium (Mg) is the lightest structural engineering material; however, the Mg alloys suffer from poor HT mechanical properties and corrosion resistance [4]. Although Mg alloys are considered for non-structural components of an aircraft like housing and casing, at present they lack the properties needed for aeroengine applications [4]. The pursuit of better alloys that are lighter than Ti has attracted significant interest, but these alternatives tend to be expensive and, therefore, not economically feasible [5,6,7,8]. Lightweight HT Al-based alloys, however, offer promise due to their cost-effectiveness, and exceptional resistance to oxidation [9]. While Al-based alloys are amongst the lightest metallic alloys, their use in aero engines is limited. For example, wrought alloys like 2xxx, 6xxx, and 7xxx series find application in aircrafts, their use in jet engines is rare due to significant strength loss after prolonged exposure to HT, for example after a duration of ~500 h in the temperature range of 100–150 °C and after ~250 h in the temperature range of 200–250 °C [2].

Alloys containing microalloying elements like Al-Mg-Mn [10] or Al-Fe-Mn [11], reinforced by dispersoids, have received limited attention due to their low solubility in Al [9,12] and subsequent inferior hardness post-aging. Conversely, alloys within the Al-Si-Cu-Mg system, microalloyed with Mo, Mn, Cr, Ti, V, and Zr, show promise due to synergistic strengthening achieved through dispersion and precipitation hardening strategies [13,14,15]. The AlCuMnZr system is gaining traction for its exceptional stability at HT and impressive mechanical properties [16]. Transition metals (TMs) play a crucial role in advancing HT Al-based alloys [9], with Al_3_TM compounds being explored as potential reinforcements due to their high melting points [9]. However, equilibrium Al_3_TM crystallizes in a body-centered tetragonal (BCT) structure. For example, Al_3_Ti and Al_3_Ta adopt the D0_22_ crystal structure [9]. Additionally, TMs in general exhibit slow diffusion in α-Al, imparting superior resistance against Oswald ripening to Al_3_TM due to volume-controlled diffusion models, thereby enhancing thermal stability and coarsening resistance of these alloys [9,17,18,19].

In this work, a novel HT Al_87.5_Ti_6.25_Ta_6.25_ (all compositions used in this paper are in at.%, unless otherwise specified) alloy was developed. The developed alloy composed of a high-volume fraction (~0.5) of hard Al_3_(Ti,Ta)-type trialuminide IP reinforced in tough Al-rich FCC matrix driven by CALPHAD in consideration for optimization of strength-toughness combination at equal volume fractions and to achieve enhanced thermal stability resulting from the elevated point of Ta and the beneficial effects of entropy stabilization leading to slow-down diffusion with the cooperative diffusion of Ti and Ta. CALPHAD method is a well-established approach to develop novel alloys in conjunction with experimental validation [20,21]. The thermodynamic calculations were performed using the thermodynamic database for Al-Ti-Ta system compiled using the work of Witusiewicz et al. [22]. This Al-Ti-Ta system is not accessed in the Thermo-Calc Al-alloy thermodynamic database (TCAL7) [23]. Furthermore, the accuracy of the thermodynamic database was investigated by comparing the thermodynamic calculations with the experiments. Additionally, the designed alloy was characterized by nanoindentation and microhardness tests to extract its mechanical properties.

## 2. Materials and Methods

In this investigation, a dual phase microstructure design strategy was pursued by targeting equimolar amounts of FCC solid solution and Al_3_TM-type IP (where TM = Ti, Ta) for reinforcement. Drawing from the established alloy design methodology [17,18,19], this paper extends its application to the Al-Ti-Ta system, specifically exploring the Al_87.5_Ti_12.5-x_Ta_x_ system (with x = 0–12.5). Leveraging a specialized CALPHAD database derived from the comprehensive thermodynamic assessment by Witusiewicz et al. [22], Thermo-Calc software 2024a (Thermo-Calc software AB, Stockholm, Sweden) [24] was employed to generate the equilibrium phase fractions at room temperature (RT), i.e., 308.15 K across the Al_87.5_Ti_12.5-x_Ta_x_ system (x = 0.25–12.5). Furthermore, equilibrium step diagram and Scheil solidification path calculations of the chosen alloy Al_87.5_Ti_6.25_Ta_6.25_ (hereby referred 3T) were performed for further understanding.

The chosen alloy, Al_87.5_Ti_6.25_Ta_6.25_ alloy, was fabricated through vacuum arc-melting, combining high-purity (99.9%) constituents sourced from Alfa Aeser at 25 g per cycle. To overcome the elemental loss, due to significantly different melting points of Al, Ti and Ta, A master alloy of (Ti,Ta) was first prepared by melting a predetermined ratio of Ti and Ta together. This master alloy was produced in a controlled environment to minimize oxidation and ensure homogeneity. Then the master alloy was melted with the predetermined Al button to attain the desired nominal alloy composition. Once the complete alloy button was obtained, it had undergone five cycles of flipping and remelting to ensure homogeneity. Subsequently using a LabSys Evo unit by SETARAM (Caluire, France), DSC analysis was performed on the 3T alloy (as-cast) to measure its melting transition temperatures and resultant isothermal heat-treatment temperature of the alloy. The DSC measurements were conducted under an inert Argon atmosphere with continuous gas flow, with the alloy heated from RT to 700 °C (Heating rate: 10 $K{min}^{-1}$). Then the alloy was stabilized for 10 min at 700 °C, followed by undergoing final stage heating at the same rate to 1500 °C. DSC data were analyzed using Calisto software from SETARAM (v2.0, Caluire, France). To attain equilibrium the as-cast buttons were isothermally heated for a period of 24 h at 475 °C in vacuum, followed by water quenching. The Archimedes method was used to measure the densities of both the as-cast and heat-treated alloys.

Phase identification on the studied alloys in both the as-cast and heat-treated conditions were performed using XRD technique. X′pert-Pro XRD unit (Malvern Panalytical, Worcestershire, UK) was used for XRD measurements by employing a Cu-K_α_ radiation (wavelength of 0.15406 nm). The XRD analysis was conducted across a 2θ scan range spanning from 20° to 90° with scan angle increment of 0.002°. This is consistent with previous studies [17,18,19]. XRD data were analyzed using X’pert Highscore plus software (v3.0, Panalytical B.V., Almelo, Netherlands). Microstructure investigations for both the alloy conditions were performed using a FEI Helios G4UX SEM (ThermoScientific, Waltham, MA, USA) having an EDS detector (Octane Elite plus). EDS calibration was perfomed using Al + Cu standard. This combination gives best calibration because AlKα and CuKα peaks are widely separated. Routine calibration is performed every 3 months to ensure accurate results. Standard metallographic procedure as described elsewhere [17,18,19], were used for SEM-EDS investigations. Image analysis was performed using ImageJ software (v1.54d, NIH, Bethesda, MD, USA).

Nanoindentation (NI) testing was further performed on the metallographically polished samples to obtain the phase-specific mechanical properties on Bruker Hysitron TI 980 TriboIndenter^®^ ( Bruker Corporation, Billerica, MA, USA) by selecting the diamond Berkovich probe (TI-0283). Standard NI calibrations were performed prior to NI tests [18,19]. The Oliver and Pharr formulation [25] was used to extract the Nanohardness (NH) and Reduced Elastic Modulus (REM) values from the NI data. A 2000 μN peak load was used during load-controlled NI tests (Loading 0.1 s, Holding 0.1 s, and Unloading 0.1 s). A total of 9 nano indents (3×3 matrix) per phase were performed using the XPM mode and the data reported in this study is derived from the mean and standard deviation of NI data. Overall hardness of the studied alloys was obtained using a Vickers micro-hardness tester (Wolpert Wilson, Buehler, Illinois, USA). A 100 g peak load with a 10 s indention holding time was used. The reported micro-hardness values result from the statistical data analysis of the 20 readings obtained from an as-cast and homogenized alloy.

## 3. Results and Discussion

### 3.1. CALPHAD-Type Calculations

**Figure 1:**
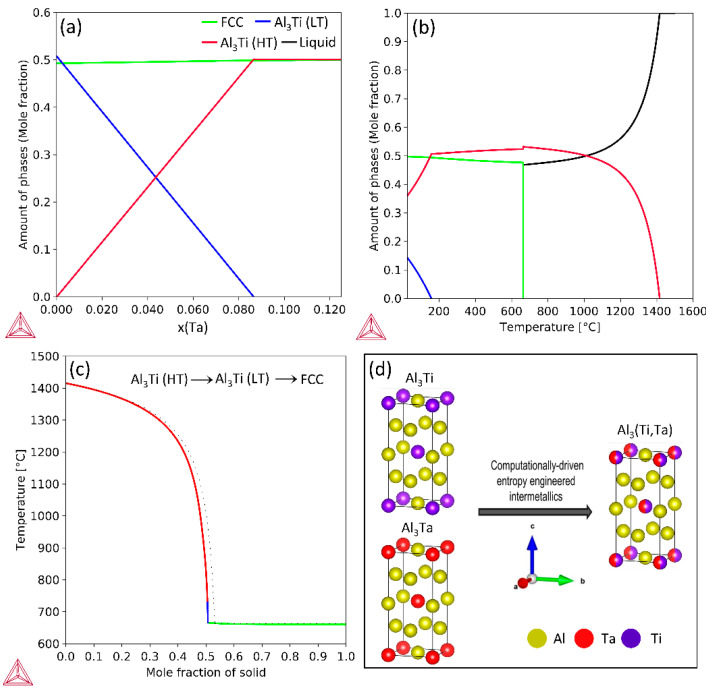
(**a**) Computed phase fraction varying with x in Al_87.5_Ti_12.5-x_Ta_x_ (x = 0–12.5). (**b**) Equilibrium step diagram for the chosen composition (x = 6.25). (**c**) Scheil solidification sequence of the designed alloy. (**d**) Crystal structure schematics of the binary D0_22_ IP (Al_3_Ti, Al_3_Ta) and entropy-engineered D0_22_-structured Al_3_(Ti,Ta) IP obtained from Vesta software [26].

At 308.15 K, the Ta concentration (x) was varied from 0 to 12.5% in the Al_87.5_Ti_12.5-x_Ta_x_ ternary system to compute the resultant phase fractions as depicted in Figure 1a. x in represents Ta concentration in atomic fraction. The solidified alloy at RT possesses three equilibrium phases, namely, an Al-rich FCC phase (solid solution) with HT BCT-D0_22_ Al_3_(Ti,Ta) IP, and LT Al_3_(Ti,Ta) IP with BCT-D0_22_ structure (LT: Low temperature). It is observed that with increasing Ta content, the LT IP phase content decreases while that of HT IP increases. The mole fraction of the FCC phase remains at nearly 0.5 throughout this composition range. Thereby the overall mole fraction of the IP remains nearly around 0.5. This calculation was performed to screen the alloy composition for further experimental exploration.

Figure 1b illustrates the equilibrium step diagram for the chosen experimental alloy: Al_87.5_Ti_6.25_Ta_6.25_. CALPHAD results reveal that the Al_3_(Ti,Ta)(LT) phase forms below 200 °C. However, in Figure 1c, Scheil simulations reveal the phase formation sequence of the designed alloy during solidification. Al_3_(Ti,Ta)(HT) IP forms first followed by a small amount of Al_3_(Ti,Ta)(LT) IP, and the FCC phase forms at the last. Table 1 lists the phase amounts obtained from CALPHAD. A detailed comparison of the thermodynamic calculations with experimentally observed data can be found in Section 3.2 and 3.3. It should be noted that the classical Schiel solidification assumes no solid-phase diffusion and infinite liquid-phase diffusion.

Although all the compositions corresponding to x(Ta) = 0–0.125, deliver 50 mol.% FCC and 50 mol.% total IP, it was anticipated that in the screened alloy composition with equal amounts of Ti and Ta, the resultant IP would benefit from entropic-phase stabilization which would promote maximum configurational disorder in the Al_3_(Ti,Ta) IP. Assuming ideal mixing in the (TM) sublattice of Al_3_(TM) IP, the configurational entropy per mole of Al_3_(Ti,Ta) IP was calculated as 0.173R (R: Universal gas constant) [27]. This entropy-driven approach [28] thereby minimizes the Gibbs free energy within Al_3_(Ti,Ta) IP leading to a thermodynamically (${∆G}_{mix}={∆H}_{mix}-T{∆S}_{mix}$) stabilized structure due to small lattice strain energy involved in the mixing (atomic radii of Ti and Ta are 14.6 and 14.7 nm, respectively), favoring the formation of ternary Al_3_(Ti,Ta) IP over binary Al_3_Ti and Al_3_Ta counterparts [29]. The Al_3_Ti, Al_3_Ta, and Al_3_(Ti,Ta) IP crystal structure schematics were visualized by employing the Vesta software [26], are depicted in Figure 1d. The preferential generation of entropy-engineered Al_3_(Ti,Ta) D0_22_ IP could be exploited to accomplish the alloy design for high phase stability and superior HT strength, owing to presence of Ta which has significantly greater melting point than Ti.

**Figure 2:**
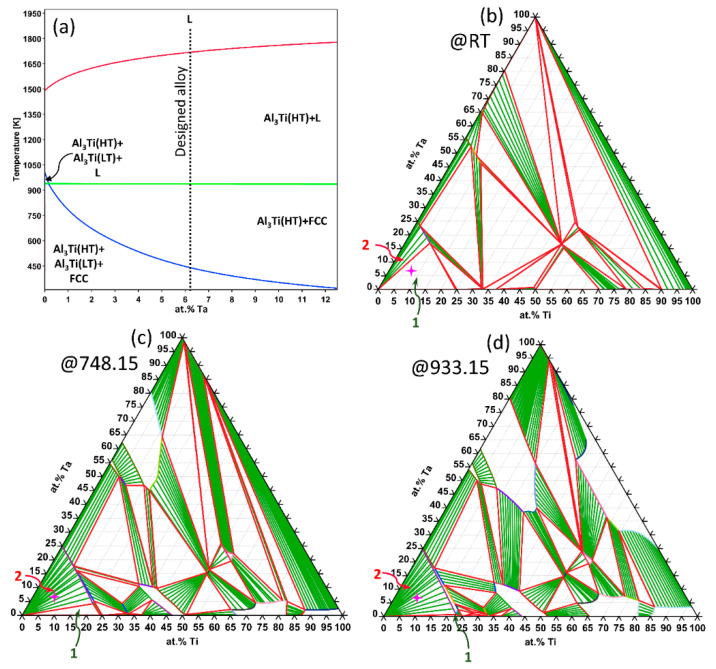
(**a**) The vertical section for the Al_87.5_Ti_12.5-x_Ta_x_ system (x = 0–12.5%). Al-Ti-Ta isothermal section obtained at (**b**) RT, (**c**) 475 °C (temperature of heat-treatment), and (**d**) 663.46 °C (Solidus point). The composition corresponding to 3T is highlighted in the isothermal section with a pink symbol. Phase region 1 is characterized by the equilibrium of Al_3_Ti(HT) + Al_3_Ti(LT) + FCC, while phase region 2 encompasses the Al_3_Ti(HT) + FCC equilibria.

Figure 2a reveals the isopleth construction of the Al_87.5_Ti_12.5-x_Ta_x_ (x = 0–12.5%) system, providing insights into the phase stability of this alloy system. It also clarifies the phase development within the Al-Ti-Ta composition space, serving as a critical resource for deciphering the alloy’s phase transformation tendencies, thereby facilitating the CALPHAD-based alloy design and entropy-driven design strategy. The diagram notably highlights the chosen alloy composition at x = 6.25%.

The isothermal sections for the Al-Ti-Ta system at RT (308.15 K), heat-treatment temperature (748.15 K), and solidus (933.46 K) are presented in Figure 2b, Figure 2c, and Figure 2d, respectively. These diagrams delineate the complex phase interactions in the alloy, particularly emphasizing the dynamic behavior of the Al_3_Ti(LT) + FCC + Al_3_Ti(HT) (Phase region 1) and Al_3_Ti(HT) + FCC (Phase region 2) across different temperatures of interest. Since the present Al-rich alloy system is located near the Al-rich corner of the composition space, only phase regions 1 and 2 are relevant. Specifically, phase region 1 contracts while phase region 2 expands with temperature increase. The selected alloy composition, Al_87.5_Ti_6.25_Ta_6.25_, is identified within the composition space of Al-Ti-Ta. These findings are in harmony with the CALPHAD analyses presented in Figure 1, collectively enhancing the understanding of the alloy system’s phase dynamics and optimizing alloy design.

Further investigation extended to Gibbs energy calculations of various phases within the Al-Ti-Ta system at 475 °C, based on the isoplethal section presented in Figure 2a. The initial calculation incorporated all the equilibrium phases viz. FCC, Al_3_Ti(HT), and Al_3_Ti(LT) into the calculation, resulting in a total Gibbs energy for the system of −42.27 kJ. The Gibbs energies for the HT-Al_3_Ti, LT-Al_3_Ti and the FCC were estimated as −56.81 kJ/mol, −27.62 kJ/mol and −56.62 kJ/mol, respectively.

The molar driving force of a phase normalized by RT (R: Universal gas constant and T: temperature (in K)) is termed as molar normalized driving force (NDF). In principle, equilibrium phases have zero driving force [30]. However, to delineate the hierarchy of phase formation in the chosen Al-Ti-Ta system it was imperative to exclude certain phases from the equilibrium computations. By omitting the Al_3_Ti(HT) and Al_3_Ti(LT) phases in the subsequent Gibbs energy calculation, NDF values of 1.15 for Al_3_Ti(HT) and 0.14 for Al_3_Ti(LT) were identified, which adjusted the system’s total Gibbs energy to −40.16 kJ. This methodological approach underscores the significance of selective phase exclusion in quantifying the driving forces critical for phase formation, offering a refined perspective on the thermodynamic stability and phase evolution within the Al-Ti-Ta system, integral to optimizing alloy design.

### 3.2. Phase and Thermal Characterization

**Figure 3:**
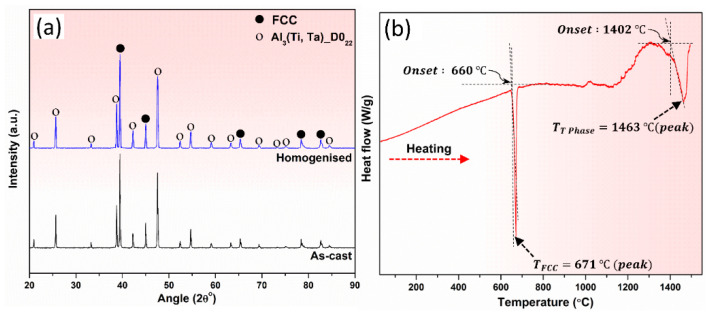
(**a**) Comparison of the XRD scans of as-cast and heat-treated alloy 3T, (**b**) DSC thermogram for as-cast alloy 3T.

Comparison between the XRD diffractograms of 3T alloy in its as-cast and heat-treated condition are presented in Figure 3a. The XRD diffractograms only reveal the presence of an FCC phase (Al-rich) and D0_22_ phase (Al_3_(Ti,Ta)) defined, herewith as T phase. The XRD-derived lattice parameters of the FCC and T phase are summarized in Table 2. It should be emphasized that lower Ti and Ta concentration is observed in the Al-rich FCC matrix in the as-cast 3T alloy as compared to the heat-treated state, this can be attributed to the larger atomic size of Ti and Ta than Al. However, in contrast, the Al_3_(Ti,Ta) IP has a higher concentration of Ti and Ta in the as-cast state as compared to the homogenized state. This has been further validated by point SEM-EDS analyses presented in this manuscript. These compositional differences have a significant role in the lattice parameter values.

In the DSC thermogram of cast 3T alloy (Figure 3b), two peaks at 671 °C and 1463 °C are observed, these represent the transition points for melting (endothermic) of FCC and Al_3_(Ti,Ta) IP, respectively. The melting initiation temperature (onset point) of FCC and T phase were computed at 660 °C and 1402 °C, respectively. Notably, the CALPHAD simulations predicted the transition points for melting of the FCC and Al_3_Ti(HT) phases as 663.2 °C and 1445.6 °C, respectively. This comparison demonstrates the close agreement between the experimentally determined melting ranges of the two phases and CALPHAD-estimated melting point data. Interestingly the melting onset temperature of the FCC matrix (Al-rich solid solution) in the designed alloy also exceeds the eutectic point of Al-Si alloys which is 577 °C. The high Al-matrix melting transition temperature in the designed alloy could have positive implications on the HT performance of the developed alloy.

To reduce microstructural segregation and heterogeneity in the as-cast alloy it was subjected to an isothermal heat-treatment at ${0.8T}_{FCC}$, equivalent to ~475 °C for 24 h. The alloy 3T’s density was experimentally determined to be 3.756 and 3.759 $g{cm}^{-3}$ for the as-cast and heat-treated condition, respectively, which lies between Al-based and Ti-based alloy systems.

### 3.3. Microstructure and Compositional Analysis

**Figure 4:**
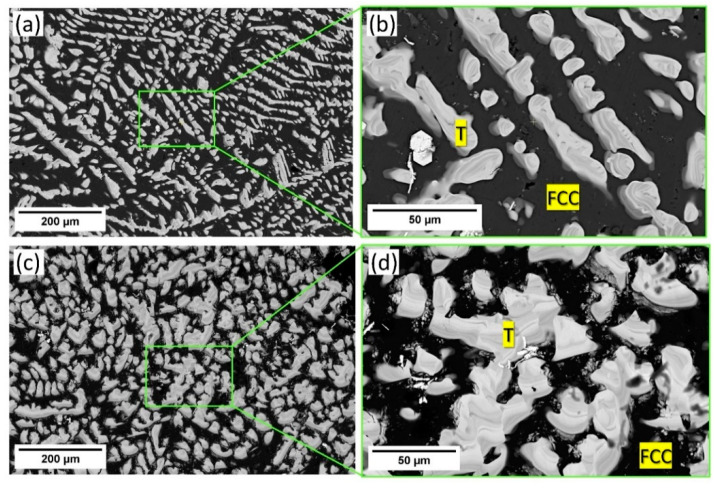
SEM-BSE micrographs in as-cast condition (**a**) Low-magnification micrograph; (**b**) High-magnification micrograph corresponding to green ROI in (**a**) and heat-treated condition (**c**) low-magnification micrograph. (**d**) High-magnification micrograph corresponding to green ROI in (**c**).

Microstructures of the as-cast (Figure 4a,b) and heat-treated alloy (Figure 4c,d) taken in SEM-BSE mode reveal a dual phase composite microstructure with black matrix (FCC) reinforced with grey secondary phases (T phase). Image analysis was performed to determine the experimental volume fraction of the phases. It was determined that the 3T alloy in as-cast and heat-treated condition had ~46 vol.% and ~52 vol.% the Al_3_(Ti,Ta) IP, respectively. Phase amountsin mole fractions obtained from thermodynamic calculations were converted to vol.% for comparison with experimental vol.% values as shown in Table 1. Furthermore, in the as-cast structure, the secondary dendrite arm spacing of the trialuminide IP measures approximately 14 μm, while the spherical IP particles exhibit a size of approximately 16 μm. This underscores the fine dendrite secondary arm spacing achieved during casting and the minimal coarsening observed during the isothermal heat-treatment, which can be attributed to sluggish cooperative diffusion.

**Figure 5:**
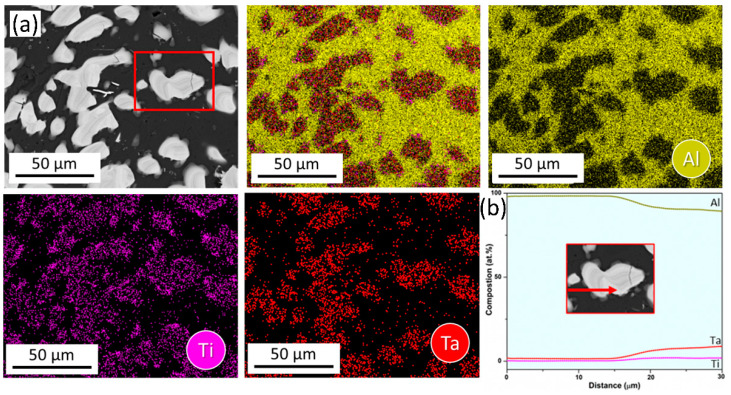
SEM-EDS analysis of as-cast 3T alloy (**a**) Elemental mapping and (**b**) Line scan along the red arrow encompassing FCC/IP interface from red ROI in (**a**).

**Figure 6:**
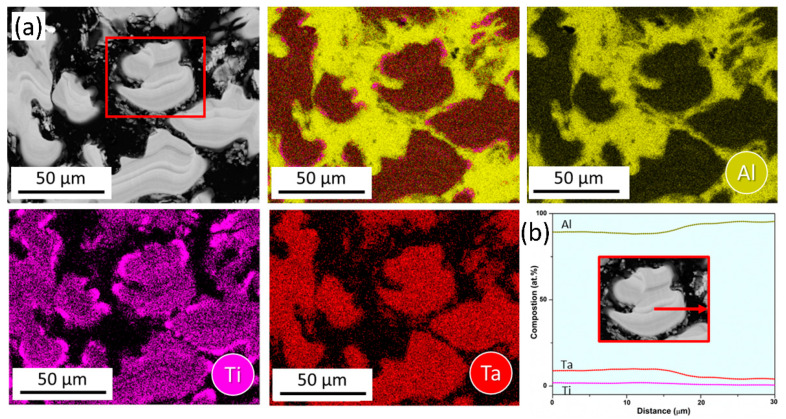
SEM-EDS analysis of heat-treated 3T alloy (**a**) Elemental mapping and (**b**) Line scan along the red arrow encompassing FCC/IP interface from red ROI in (**a**).

The SEM-EDS bulk composition analyses of both the as-cast and heat-treated alloy 3T (Table 3), indicates negligible deviation between the experimental and theoretical alloy-design compositions with no interstitial impurities, and a comparison between the experimentally measured phase compositions of both the conditions with CALPHAD-derived phase compositions is presented in Table 4. The results confirm that the FCC phase represents the Al-TM solid solution (Al-rich), and the T phase is Al_3_TM-type entropy-stabilized ternary IP (TM = Ti, Ta). SEM-EDS elemental maps and line scan for both conditions as presented in Figure 5 and Figure 6**,** respectively, and these support the previous observation.

### 3.4. Mechanical Properties

**Figure 7:**
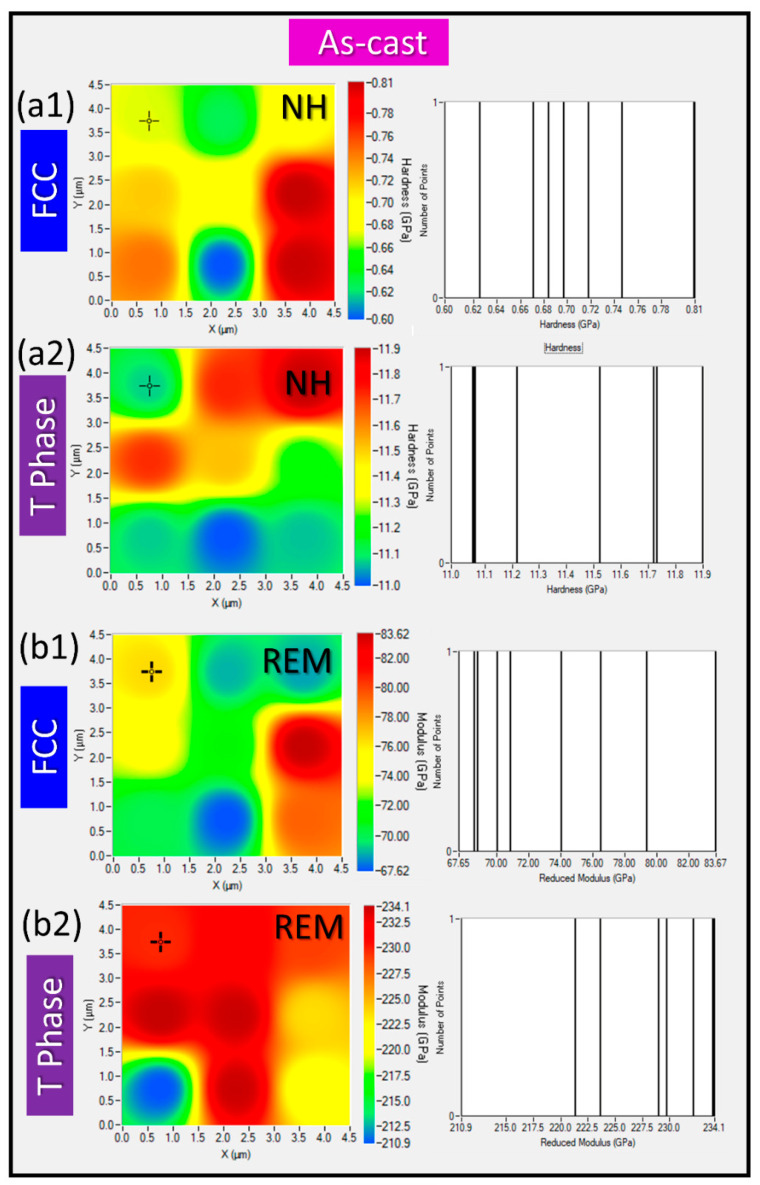
NI data for 3T alloy in its as-cast state with NI-XPM map for the FCC and T phase in (**a1**) and (**a2**), respectively, and REM-XPM maps for the FCC and T Phase are presented in (**b1**,**b2**).

**Figure 8:**
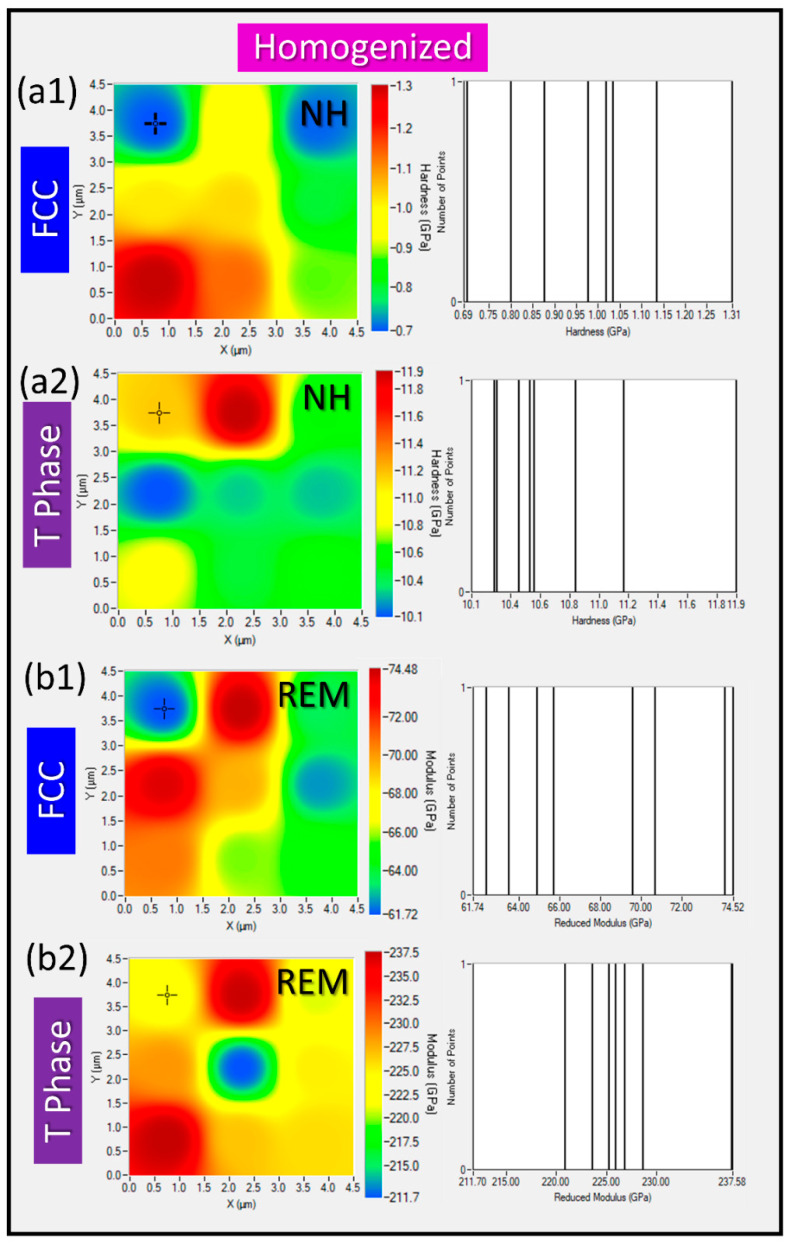
NI data for 3T alloy in its as-homogenized state with NI-XPM map for the FCC and T phase in (**a1**) and (**a2**), respectively, REM-XPM maps for the FCC and T Phase are presented in (**b1**,**b2**).

NI with Accelerated Property Mapping (XPM) was used to measure nanohardness (NH) and reduced elastic modulus (REM) across specific phases within an area of interest. XPM enables rapid data acquisition by performing high-density indent arrays, significantly speeding up the mapping process compared to conventional NI. Thus, through high-throughput spatial maps for NH and REM, variation of nanomechanical properties across different phases was studied. XPM efficiently captures data variation across a dense grid of points, providing better statistical confidence and revealing localized variations in mechanical properties. A phase-specific map of NH and REM for alloy 3T in as-cast condition is presented in Figure 7, while a phase-specific map of NH and REM for alloy 3T in heat-treated condition is presented in Figure 8. The phase-specific NI results of the as-cast alloy are: ${NH}_{FCC}=0.71\pm 0.06\;GPa;{REM}_{FCC}=73\pm 5\;GPa;{{NH}_{TPhase}=11.4\pm 0.3\;GPa;REM}_{TPhase}=228\pm 7\;GPa,$ while the phase-specific NI results of the homogenized alloy are: ${NH}_{FCC}=0.95\pm 0.09\;GPa;{REM}_{FCC}=68\pm 5\;GPa;{{NH}_{TPhase}=10.9\pm 0.5\;GPa;REM}_{TPhase}=226\pm 7\;GPa.$ These, NI-XPM results provide a well-correlated phase-property relationship.

**Figure 9:**
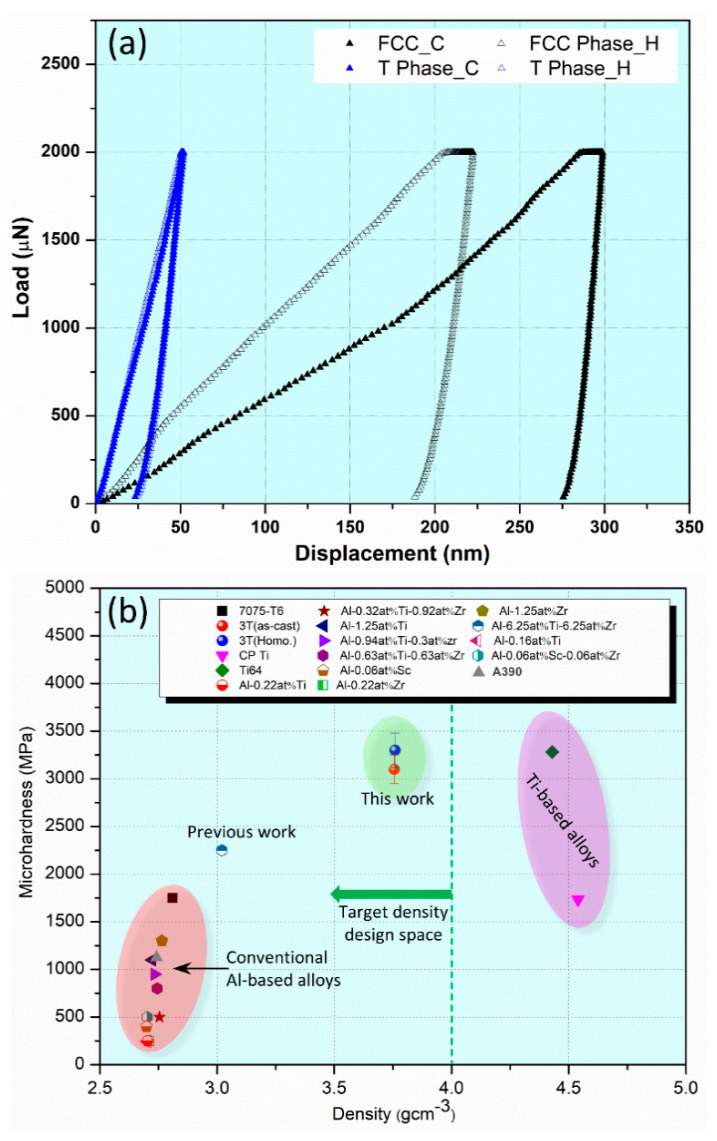
(**a**) Representative NI load-displacement curves of the as-cast and homogenized alloy obtained from both FCC and T phase. (**b**) Microhardness vs. density Ashby-type map highlighting the outstanding potential of the 3T alloy in comparison to traditional Al alloys [31,32], cast Al-Si alloy (A390) [33], CP-Ti and Ti6Al4V alloy [34].

It should be noted that NH of the FCC phase is enhanced after heat treatment, while the NH of the T phase remains unchanged. The previous observation is further validated by the superimposed load-displacement curves obtained during NI as presented in Figure 9a. In the FCC phase, the incorporation of higher content of Ti and Ta into the Al matrix (Table 4) enhances solid solution strengthening. This occurs because solute atoms create lattice distortions that impede dislocation movement, leading to increased NH. However, the nanomechanical properties of T phase remain unchanged even after 24 h of exposure at 475 °C. The T phase demonstrates remarkable thermal stability, maintaining its nanomechanical properties even after prolonged exposure at HT. This stability ensures that the T phase continues to contribute to the alloy’s overall hardness without undergoing softening, effectively acting as a reinforcing phase alongside the FCC phase.

The overall microhardness of as-cast and heat-treated alloy was found to be 3100±150 and 3300±180 MPa, respectively. The increased overall hardness after heat-treatment is consistent with NI results. The interaction between the hard FCC phase and the stable T phase allows for effective load transfer during deformation. When the FCC phase yields under stress, the T phase can help distribute the load, enhancing the overall mechanical performance. Thus, the increased microhardness after heat-treatment is due to the coupled effect of the hardened FCC phase and thermally stable T phase. It is evident that the present alloy clearly outperforms the traditional Al-based alloys [31,32], Al-Si cast alloy (A390) [33], CP-Ti and Ti-6Al-4V alloy [34] as demonstrated in the established microhardness vs. density Ashby-type map [35] as shown in Figure 9b. At an exceptional microhardness of 3300 MPa in the designed alloy 3T exceeds the microhardness levels of 7075-T6 alloy, cast A390 eutectic alloy (Al-Si), CP-Ti, and Ti6Al4V alloy.

## 4. Conclusions

This study highlights the successful development of a lightweight Al_87.5_Ti_6.25_Ta_6.25_ (at.%) alloy using the CALPHAD method and an entropy-driven design approach, validated by experimental results. The alloy, with a density of 3.75 g/cm^3^, contains ~50 vol.% in-situ formed Al_3_(Ti,Ta) ternary trialuminide reinforcements. Thermodynamic predictions by Witusiewicz et al. [22] guided the alloy selection, revealing an Al-rich FCC phase and entropy-stabilized Al_3_(Ti,Ta)(HT) phase, with a high melting onset temperature of 660 °C, surpassing the typical eutectic point of cast A390 alloys. Post-heat treatment, the alloy demonstrated improved NH in the FCC phase due to enhanced solid solution strengthening and overall enhanced microhardness of around 3300 MPa, comparable to Ti-6Al-4V alloy. The hardened FCC and thermally stable IP are responsible for enhanced properties of the developed alloy. This research exemplifies the effective combination of CALPHAD predictions and entropy-driven design in creating advanced, thermally stable lightweight alloys, offering a promising strategy for advanced Al-alloy development for HT applications.

## Figures and Tables

**Table 1:** Quantitative comparison of phase amounts derived from SEM image analysis and CALPHAD predictions (in mol.%).

	SEM Image Analysis		CALPHAD Predictions	
	FCC (Al-Rich Black Phase)	T Phase (Al_3_(Ti,Ta) Bright Phase)	FCC	Al_3_(Ti,Ta) (HT)
As-cast	53.3 ± 1.4 54.2 ± 0.9 ^@^	47.1 ± 1.8 46.1 ± 1.1 ^@^	49.3 ^#^	50.7 ^#^
Heat-treated	49.1 ± 1.3 48.2 ± 1.1 ^@^	50.9 ± 1.3 52.3 ± 1.4 ^@^	49.9^*^	50.1 *

^#^: Classical Scheil simulations @ Solidus ~ 663.46 °C. *: Equilibrium thermodynamic predictions at 475 °C ($0.8T_{FCC})$. ^@^: Experimentally determined vol.% of the constituent phases from image analysis. (other values represent phase amounts in mole percentages).

**Table 2:** Lattice parameters of the constituent phases in as-cast and homogenized 3T alloy obtained from XRD data.

Condition	FCC	T Phase
As-cast	a=0.4042 nm	a,b=0.3784 nm;c=0.8372 nm
Heat-treated	a=0.4048 nm	a,b=0.3781 nm;c=0.8371 nm

**Table 3:** Bulk compositional analysis (n = 5) of the designed alloy 3T (at.%).

Sample Condition	Composition Type	Al	Ti	Ta
As-cast	Design	87.5	6.25	6.25
	Experimental	88.3 ± 1.1	5.9 ± 0.5	6.2 ± 0.6
Heat-treated (@475 °C for 24 h)	Design	87.5	6.25	6.25
	Experimental	87.8 ± 1.4	5.9 ± 0.4	6.2 ± 0.5

**Table 4:** Experimental (n = 5) and computed phase compositions in at.%.

Condition	Phases	Composition		
		Al	Ti	Ta
As-cast	Black (FCC)	98.0 ± 0.9	1.5 ± 0.2	0.5 ± 0.1
	Grey (T Phase)	77.5 ± 0.6	10.1 ± 0.4	12.4 ± 0.5
Heat-treated (475 °C, 24 h)	Black (FCC)	97.1 ± 0.8	1.8 ± 0.4	1.0 ± 0.2
	Grey (T Phase)	77.9 ± 0.8	10.1 ± 0.7	12.2 ± 0.4
Scheil Simulation @ 663.46 °C	FCC	99.56	0.41	0.03
	Al_3_(Ti,Ta)(HT)	75.7	11.9	12.4
Equilibrium Calculation @ 475 °C	FCC	99.81	0.17	0.02
	Al_3_(Ti,Ta)(HT)	75.26	12.45	12.29

## Data Availability

All data to evaluate the conclusions are presented in the manuscript.
